# Broad Spectrum Deubiquitinase Inhibition Induces Both Apoptosis and Ferroptosis in Cancer Cells

**DOI:** 10.3389/fonc.2020.00949

**Published:** 2020-06-12

**Authors:** Li Yang, Xin Chen, Qianqian Yang, Jinghong Chen, Qingtian Huang, Leyi Yao, Ding Yan, Jiawen Wu, Peiquan Zhang, Daolin Tang, Nanshan Zhong, Jinbao Liu

**Affiliations:** ^1^The Department of Physiology, School of Basic Medical Sciences, Guizhou Medical University, Guizhou, China; ^2^Guangzhou Municipal and Guangdong Provincial Key Laboratory of Protein Modification and Degradation, State Key Lab of Respiratory Disease, School of Basic Medical Sciences, Guangzhou Medical University, Guangzhou, China; ^3^Translational Medicine Center, The Second Affiliated Hospital, Guangzhou Medical University, Guangzhou, China; ^4^Department of Surgery, UT Southwestern Medical Center, Dallas, TX, United States

**Keywords:** deubiquitinase, apoptosis, ferroptosis, GPX4, cancer

## Abstract

Proteasomal deubiquitinase (DUB) inhibition has been found to be effective in experimental cancer therapy by inducing proteasome inhibition and apoptosis. Ferroptosis is a form of regulated cell death characterized by an iron-dependent lipid peroxidation. Antioxidant enzyme glutathione peroxidase 4 (GPX4) plays a key role in blocking ferroptosis through directly reducing phospholipid hydroperoxides production. Since cytoplasmic DUB inhibition can promote protein degradation in the cell, we hypothesize that DUB inhibition induces GPX4 degradation. Here we used palladium pyrithione complex (PdPT), a broad spectrum deubiquitinase inhibitor, to explore its cell death induction and anti-cancer effect *in vitro, ex vivo, and in vivo*. Mechanically, caspase activation and GPX4 protein degradation are required for PdPT-induced apoptosis and ferroptosis, respectively. Notably, PdPT-induced multiple deubiquitinase inhibition is essential for proteasomal degradation of GPX4. These findings not only identify a novel mechanism of post-translational modification of GPX4 in ferroptosis, but also suggest a potential anti-caner therapeutic strategy using Pan-DUB inhibition.

## Introduction

Cancers can occur when the balance of cell growth and cell death is disturbed. Thus, one of the goals of cancer therapy is to induce various types of regulated cell death or overcome drug resistance. In particular, induction of ferroptosis, an iron-dependent regulated necrosis, by small molecules (e.g., erastin and RSL3) or drugs (e.g., sulfasalazine and sorafenib), is becoming an attractive anticancer strategy (1–5). While the exact molecular mechanism of ferroptosis remains unclear, the glutathione (GSH)-dependent antioxidant enzyme glutathione peroxidase 4 (GPX4) is presumed to play a central role in the blocking ferroptosis (2). Excessive iron accumulation could lead to reactive oxygen species (ROS) production and subsequent ferroptosis through induction of intracellular lipid peroxidation (6). In contrast, GPX4 can remove the dangerous products of iron-dependent lipid peroxidation and therefore protect cell membrane against ferroptosis (7). Consequently, the GPX4 inhibitor such as RSL3 and FIN56 can trigger the ferroptotic cell death through activation of lipid peroxidation (2, 8). Thus, understanding the transcriptional and post-translational modifications of GPX4 in ferroptosis may facilitate the development of a new type of anticancer agents.

The ubiquitin-proteasome system (UPS) was discovered in the 1980s and is an evolutionarily conserved mechanism of protein degradation and turnover (9). This pathway typically consists of three components, including the ubiquitin-conjugating system, the deubiquitinating enzymes (DUBs), and the proteasome (10). The human genome encodes ~100 DUBs and further divides into six families, according to their catalytic and structural features (11). Five families of DUBs are cysteine proteases including ubiquitin C-terminal hydrolases, ubiquitin specific proteases, ovarian tumor proteases, Machado-Josephin domain proteases, and monocyte chemotactic protein-induced proteins (11). The sixth family is termed JAB1/MPN/Mov34 metalloenzyme, a type of zinc-dependent metalloprotease (11). The major function of DUBs is to cleave ubiquitin moieties from target proteins or polyubiquitin chains prior to their degradation (12). Dysfunction of DUBs is implicated in multiple cellular processes and human diseases, including cancer (11, 13). Despite the remarkable success of proteasome inhibitor in certain cancer therapies, the optimal use of proteasome inhibitors as a cell death inducer continues to be an active area of research.

The biologically active metal-based compounds such as cisplatin and oxaliplatin are of interest in both prevention and treatment of cancer (14). We have previously demonstrated that certain metal-based compounds can suppress tumor growth through targeting DUBs such as USP14 and UCHL5 to induce apoptosis (15). A series of metal-based pyrithione compounds were synthesized and various metal compound exhibits different characteristics (15, 16). In this study, we provide the first evidence that palladium pyrithione complex (PdPT), a pan-inhibitor of DUBs, not only cause apoptosis, but also induce ferroptosis to suppress tumor growth *in vitro* and *in vivo*. At the molecular level, we further demonstrate that PdPT promotes ferroptosis through proteasomal degradation of GPX4 *via* deubiquitinating of GPX4. These findings not only identify a novel mechanism of post-translational modification of GPX4 in ferroptosis, but also suggest that certain DUBs are attractive drug targets in cancer therapy.

## Materials and Methods

### Reagents

PdPT was synthesized in our laboratory and dissolved in dimethyl sulfoxide (DMSO) to a concentration of 10 mM and stored at −20°C. Bortezomib (2204) was purchased from Cell Signaling Technology (Beverly, MA, USA). b-AP15 (662140), N-Ethylmaleimide (E1271), cisplatin (#232120), and DMSO (D2650) were purchased from Sigma-Aldrich (St Louis, MO, USA). RSL3 (S8155), ferrostatin-1 (S7243), deferoxamine (S5742), and Z-VAD-FMK (S7023) were purchased from Selleckchem (Houston, TX, USA). 20S and 26S human proteasome preparation (E-350 and E-365), Suc-Leu-Leu-Val-Tyr-aminomethylcoumarin (Suc-LLVY-AMC, S-280), HA-Ubiquitin-Vinyl Sulfone (HA-Ub-VS, U-212), ubiquitin-AMC (U-550) were purchased from Boston Biochem (Cambridge, MA, USA). Anti-ubiquitin (sc-8017) was purchased from Santa Cruz Biotechnology (Santa Cruz, CA, USA). Anti-caspase-3 (9665), anti-caspase-8 (9746), anti-caspase-9 (9508), anti-PARP (9542), cleaved caspase-3 (9661), cleaved caspase-8 (9496), cleaved caspase-9 (9501), anti-K48-ub (8081), anti-HA-tag (3724), anti-USP14 (11931), anti-USP15 (66310), anti-USP10 (8501), and anti-USP7 (4833) were purchased from Cell Signaling Technology (Beverly, MA, USA). Anti-USP25 (ab187156), anti-OTUB1 (ab175200), anti-OTUD1 (ab122481), anti-UCHL5 (ab133508), and anti-GPX4 (ab16739) were purchased from Abcam (Cambridge, MA, USA). GAPDH (BS60630) was purchased from Bioworld Technology (St. Louis Park, MN, USA). Immunoprecipitation assay kit (14311D) was obtained from Life Technologies (Carlsbad, CA). Annexin V-fluoroisothiocyanate (FITC)/propidium iodide (PI) apoptosis detection kit (KGA108) were purchased from Keygen Company (Nanjing, China). Enhanced chemiluminescence (ECL) reagents (sc-2048) were purchased from Santa Cruz Biotechnology (Santa Cruz, CA, USA).

### Cell Line and Cell Cultures

The NSCLC cell line A549 was purchased from ATCC (Manassas, VA, USA) and NCI-H1299 was purchased from the Cell Bank of Shanghai Institutes for Biological Sciences, Chinese Academy of Sciences (Shanghai, China). A549/DDP and human bronchial epithelial BEAS-2B were gift from Dr. Z. He and Dr. B. Li. All cell lines were cultured in RPMI 1640 medium (Hyclone, Logan, UT, USA) supplemented with 10% fetal bovine serum (FBS) (Gibco-Invitrogen, Carlsbad, CA, USA), 0.1% of P/S antibiotic (100 U/mL penicillin, 0.1 mg/mL streptomycin; Gibco). A549/DDP cells were routinely maintained in the same medium in the presence of 1.5 μg/mL cisplatin, which was removed before experiments were started with a washout period of 2–3 days. All cells were maintained in a humidified incubator at 37°C, in the presence of 5% CO_2_.

### Cell Viability Assay

Cell viability was evaluated with MTS assay (CellTiter 96 Aqueous One Solution reagent; Promega, Shanghai, China). Briefly, A549, NCI-H1299, A549/DDP and BEAS-2B cells were seeded into 96-wells plate at a density of ~5,000 cells per well and incubated in RPMI-1640 medium with 10% FBS in a final volume of 100 μL overnight. After treatment with increasing concentrations of PdPT for 24 and 48 h, 20 μL MTS was added to each well and cells were incubated for another 3 h. Cisplatin (0, 1.25, 2.5, 5.0, and 10 μM) or DMSO alone was used as control. Absorbance was measured at wavelength 490 nm. Cell viability was expressed as a percentage of control cells and the concentration of drug required to obtain 50% inhibition in cell viability was determined as IC_50_. IC_50_ values were calculated by GraphPad Pro Prism 5.0 (GraphPad, San Diego, CA).

### Cell Death Assay

Cell death was determined using AnnexinV-FITC / PI apoptosis detection kit. A549 and NCI-H1299 cells were seeded in 6-cm dishes overnight in RPMI 1640 medium supplemented with 10% FBS, then indicated treatments with PdPT for 24 h, and the cells were digested by trypsin and washed twice with ice-cold PBS. The cell pellet was suspended with a working solution (500 μl binding buffer with 5 μl Annexin V-FITC) for 15 min in the dark at room temperature. Cells were washed and resuspended with binding buffer. PI was added just before flow cytometric analysis. Annexin V/PI staining was also imaged using an inverted fluorescence microscopy equipped with a digital camera (AxioObsever Z1, Zeiss, Germany).

### Western Blot Analysis

Western blot was performed to analyze protein expression as we previously described (16). In brief, an equal amount of the total protein extracted from cell lysates was fractionated by 12% SDS- PAGE and transferred to polyvinylidene difluoride (PVDF) membrane filters. The membranes were blocked with 5% non-fat milk in Tris-buffered saline containing 0.1% Tween 20 (TBS-T) for 60 min at room temperature. Incubations with primary antibodies (1:1,000) were carried out in TBS-T overnight at 4°C. After incubation with peroxidase-conjugated secondary antibodies (1:5,000; 1 h), the signals were detected by the ECL detection reagents according to the manufacturer's instructions and exposed to X- ray films (Kodak, Rochester, NY, USA).

### Sample Collection and Cell Culture

The use of human samples is approved by the Institution with the permission of the patients and volunteers. Six patients with AML and six healthy volunteers were recruited in this preclinical study. Bone marrow and blood samples of patients with AML were obtained from the Department of Hematology (Second Affiliated Hospital, Guangzhou Medical University). Peripheral blood samples of normal control individuals were obtained from Guangzhou Blood Center. Ficoll-Paque (Pharmacia, Uppsala, Sweden) was used to isolate mono-nuclear cells from either peripheral blood or bone marrow samples by density gradient. The mononuclear cells were cultured in RPMI 1640 culture medium with 15% FBS as described previously (16).

### Peptidase Activity Assay

Chymotrypsin-like activity of the proteasome was assessed with fluorogenic Suc-LLVY-AMC substrate. To assay for *in vivo* proteasome inhibition, non-small cell lung cancer cells were treated with PdPT or bortezomib as a positive control for 4 h. The cells were lysed in ice-cold lysis buffer. Equal amounts of protein from each sample were then incubated at 37°C with 50 μM fluorogenic substrates. To assay for direct inhibition of the 20S proteasome *in vitro*, purified human 20S proteasomes were incubated with the agent to be tested for 60 min at 37°C before the addition of the fluorogenic substrate. Fluorescence intensity was measured using a spectrophotometer at excitation of 350 nm and emission of 438 nm (Varioskan Flash 3001, Thermo, Waltham, MA, USA).

### DUB Activity Assay

DUB activity assay was performed as reported previously (16). Briefly, A549 and NCI-H1299 cells were incubated with different concentration PdPT (2.5 or 25 μM), and b-AP15 (25 μM) alone for 6 h. These cells were dissolved in ice-cold DUB buffer containing 50 mM Tris-HCl (pH 7.4), 20 mM NaCl, 5 mM MgCl_2_ and 200 μM ATP. An equal amount of the total protein extracted from cell lysates was incubated with or without HA-UbVS substrate in 37°C for 0.5 h. Then, the protein samples were used to do Western blot analysis.

### Immunohistochemical Staining

Formalin-fixed xenografts were embedded in paraffin and sectioned according to standard techniques as we previously reported (17). Tumor xenograft sections (4 μm) were immunostained using the MaxVision kit (Maixin Biol, Fuzhou, China) according to the manufacturer's instructions. The primary antibodies were used as indicated. In all, 50 μl MaxVisionTM reagent was applied to each slide. Color was developed with 0.05% diaminobenzidine and 0.03% H_2_O_2_ in 50 mM Tris-HCl (pH 7.6), and the slides were counterstained with hematoxylin. A negative control for every antibody was also included for each xenograft specimen by substituting the primary antibody with preimmune rabbit serum. The stained tumor sections were examined independently by two observers for positively stained tumor cells and the intensity of immunohistochemical signals. The integreted optical density (IOD) value and Aera value determined by Image-Pro Plus software. IOD/area is average optical density(AOD).

### Protein Interaction Analysis

Immunoprecipitation analysis was performed as described in a previous study (18). In brief, antibodies and dynabeads (Invitrogen) mixtures were prepared overnight. Then the cell lysates extracted from A549 and NCI-H1299 cells were added in the antibodies-beads mixtures. After incubation and rotation at 4°C for 1 h, the antibodies-prays mixtures were washed with PBS-T for three times. Then the mixtures were suspended with appropriate SDS loading buffer and separated from dynabeads. Western blot was used to analyze protein expression. This assay was performed as described previously.

### Nude Mouse Xenograft Model

All animal protocols used were approved by the Institutional Animal Care and Use Committee of Guangzhou Medical University (Approval No. GY 2019–113). The nude BALB/c mice (male, 5-week-old, 18–22 g) were purchased from Guangdong Medical Laboratory Animal Center (Foshan, China) and housed in barrier facilities with a 12 h light dark cycle, with food and water available *ad libitum*. 1–10 × 10^6^ of NCI-H1299 or A549 cells in 200 μl phosphate-buffered saline were injected subcutaneously to the right flanks of nude mice. After 3 days of inoculation, mice were treated with either vehicle (10% DMSO, 30% polythylene glycol 400, and 60% 0.9% NaCl) or PdPT (7.5 mg/kg/day) for 2 weeks (7 intervals). Tumor volumes were recorded and calculated as previously reported (17). On day 15 after the start of treatment, tumor xenografts were removed, weighed, stored, and fixed. All experiments were performed in accordance with relevant guidelines and regulations.

### Statistical Analysis

The data were expressed as Mean ± SD where applicable. GraphPad Prism 4.0 software (GraphPad Software) was used for statistical analysis. Student's *t*-test or one way ANOVA was used to compare the differences between variables. *P*-value of < 0.05 was considered statistically significant.

## Results

### PdPT Is an Inhibitor of Multiple DUBs

**Figure 1:**
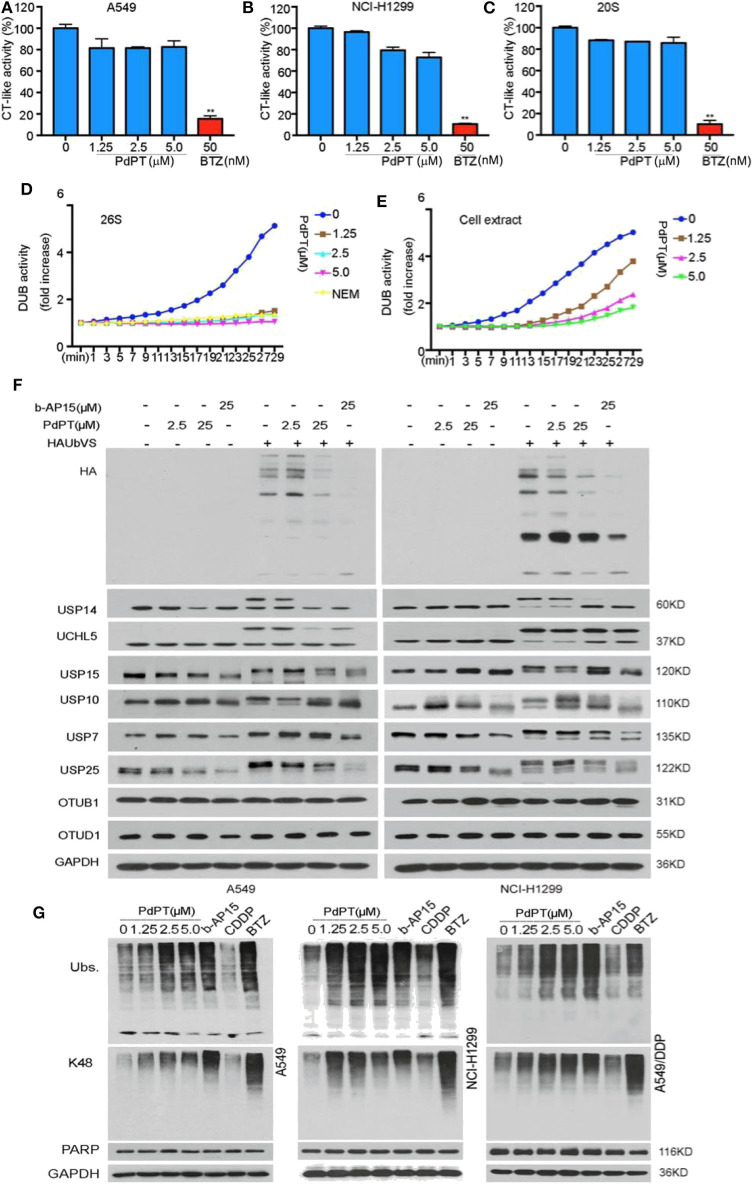
PdPT is an inhibitor of multiple DUBs. a and b The *in situ* and *in vitro* effects of PdPT on proteasome peptidase activity. Lysates were from PdPT or BTZ-treated A549 **(A)** and NCI-H1299 **(B)**. Cells were treated with the indicated doses of PdPT and then the proteasome chymotrypin-like activity were analyzed. **(C)** Purified 20S proteasomes were treated with the indicated doses of PdPT and then the chymotrypin-like activity was measured using specific synthetic fluorogenic substrates. BTZ was used as a positive control. Values are expressed as mean ± SD (*n* = 3). **P < 0.01, compared with each control. d and e The effect of PdPT on DUB activities. Purified 26S proteasomes **(D)** or A549 cell lysates **(E)** were exposed to PdPT (1.25, 2.5, and 5.0 μM) and the dynamic DUB activity was measured. NEM was used as a positive control. **(F)** Abolishment of Ub-VS labeling of proteasomal DUBs by PdPT. Lysates from PdPT (2.5, 25 μM) or b-AP15 (25 μM)-treated A549 and NCI-H1299 cells were incubated with labeled HA-UbVS at 37°C for 30 min. The levels of HA and DUBs were assayed using western blot. **(G)** A549, NCI-H1299, and A549/DDP cells were treated with indicated PdPT for 6 h. Total ubiquitinated proteins (Ubs.), K48-linked ubiquitinated proteins (K48), and PARP proteins were detected using western blot. Bortezomib(BTZ, 100 nM) and b-AP15 (1 μM) were used as a positive control and CDDP (10 μM) was used as a negative control.

To identify the target of PdPT, we first tested 20S proteasome peptidase activities in two human non-small cell lung cancer (NSCLC) cell lines (A549 and NCI-H1299). Biochemical assays found that 20S chymotrypsin-like peptidase activities were not significantly affected in A549 and NCI-H1299 cell lines in response to PdPT (Figures 1A,B). PdPT also failed to diminish the activities of the purified 20S proteasome chymotrypsin-like peptidases in a cell free system (Figure 1C). As expected, bortezomib, the first therapeutic proteasome inhibitor, blocked 20S proteasome peptidase activities in these cell lines or cell free systems. These findings indicate that PdPT may not be a direct inhibitor of 20S proteasome.

We next assayed whether PdPT controlled DUB activities. Indeed, PdPT dose-dependently inhibited DUB activities in either purified 26S proteasomes (Figure 1D) or cell lysates (Figure 1E). Consistent with previous studies (16), N-ethylmaleimide (NEM), a general inhibitor of cysteine proteases, also diminished DUB activities in either purified 26S proteasomes (Figure 1D) or cell lysates (Figure 1E). These findings indicate that PdPT has similar activity as NEM to block DUB.

To further identify the direct target of PdPT in proteasome-associated DUBs, we performed competitive labeling experiments using HA-Ubiquitin-Vinyl Sulfone (HA-Ub-VS), a DUB active site directed probe that generated by a chemical ligation method (19). PdPT (2.5 and 25 μM) was able to compete with HA-Ub-VS for binding to USP14, UCHL5, USP15, USP10, USP7, and USP25 like b-AP15, but not OTUB1 and OTUD1 (Figure 1F). Like bortezomib and b-AP15 (a deubiquitinase inhibitor for 19S proteasomes activity), PdPT exhibited similar activity in the up-regulation of the total and the K48-linked ubiquitinated protein expression in A549, NCI-H1299, as well as cisplatin-resistance cell line A549/DDP. In contrast, cisplatin failed to affect expression of total and the K48-linked ubiquitinated protein (Figure 1G). Collectively, these findings suggest that PdPT is a potential inhibitor of multiple DUBs.

### Anti-cancer Activity of PdPT *in vitro*

**Figure 2:**
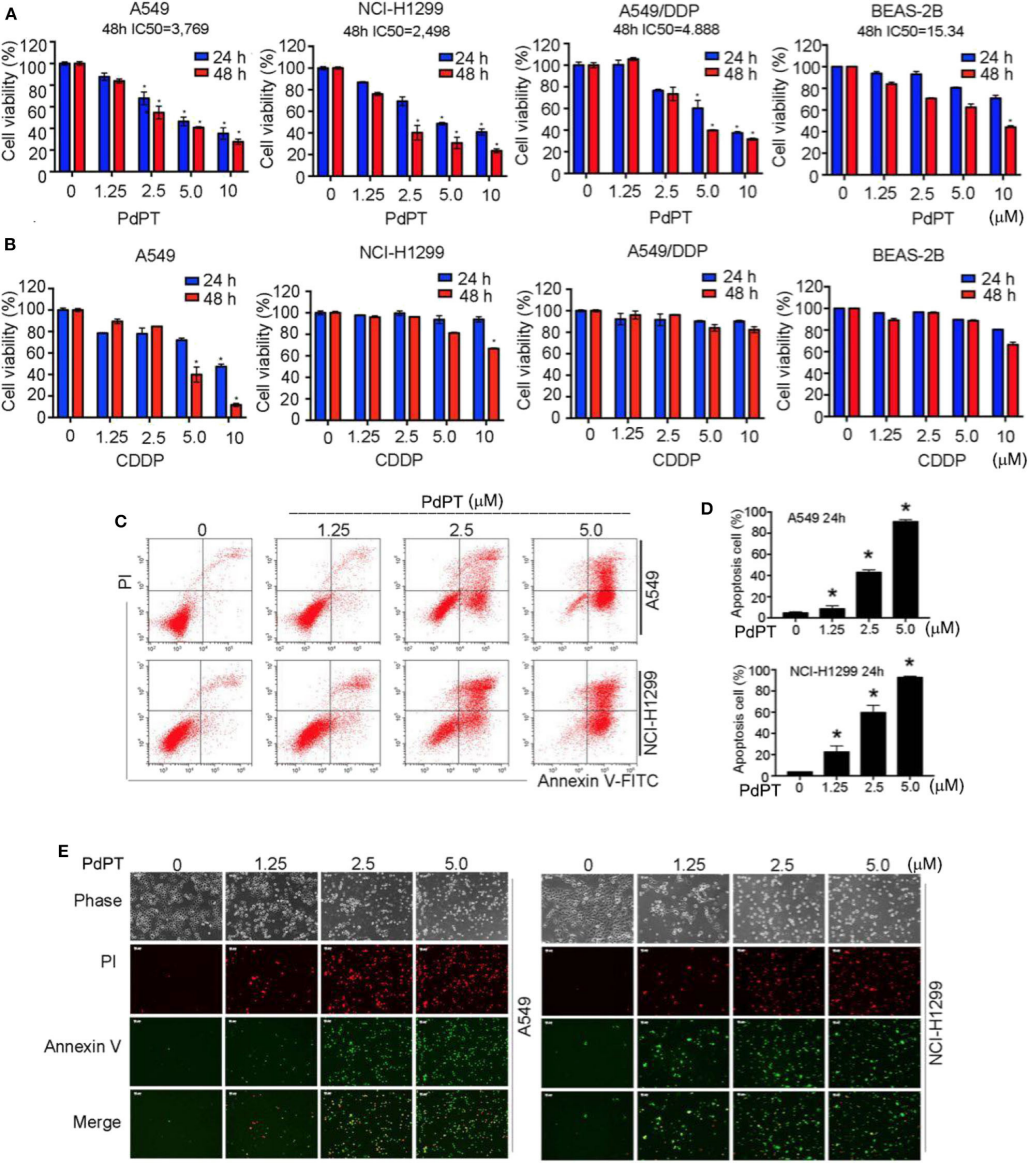
Anti-cancer activity of PdPT *in vitro*. **(A)** A549, NCI-H1299, A549/DDP and BEAS-2B cells were exposed to indicated concentration of PdPT for 24 or 48 h. Cell viability was detected by MTS assay (*n* = 3; *P < 0.05). **(B)** A549, NCI-H1299, A549/DDP and BEAS-2B cells were exposed to indicated CDDP for 24 or 48 h. Cell viability was detected by MTS assay (*n* = 3; *P < 0.05). **(C,D)** A549 and NCI-H1299 cells were treated with indicated PdPT for 24 h. Apoptotic cells were detected with Annexin V-FITC and PI double stain followed by flow cytometry. **(E)** In parallel, fluorescence microscope were used to monitor the levels of Annexin V- or PI-positive cells.

Given that many DUBs inhibitors are cell death inducers (20), we determined whether PdPT could suppress tumor cell growth by its direct cytotoxic effects. Cancer cell lines (A549, NCI-H1299 and A549/DDP) and human bronchial epithelial BEAS-2B cells were treated with PdPT (1.25–10 μM) for 24 and 48 h and then cell viability was assayed by MTS kits. Indeed, PdPT significantly resulted in cell growth inhibition in a dose- and time-dependent manner. The IC_50_ of PdPT at 48 h was 3.769, 2.498, 4.888, and 15.34 μM in A549, NCI-H1299, A549/DDP, and BEAS-2B cells, respectively (Figure 2A). Furthermore, PdPT exhibited higher anticancer activity in these cell lines compared to CDDP at the same concentrations (Figure 2B).

To determine whether PdPT-mediated growth inhibition is due to induction of cell death, we performed both flow cytometry and fluorescent microscope analysis using annexin V and propidium iodide (PI) double staining. Annexin V can bind to phosphatidylserine (PS) in early apoptotic cells when PS translocates to the external leaflet (21). In contrast, PI can bind to intracellular DNA when plasma membrane is ruptured during necrosis (22). Notably, the percentage of annexin V- or PI-positive cells was significantly up-regulated in A549 and NCI-H1299 cell lines following PdPT treatment (Figures 2C–E). Together, these findings suggest that PdPT induces apoptosis and necrosis in cancer cells.

### Caspase Activation Is Required for PdPT-Induced Apoptosis

**Figure 3:**
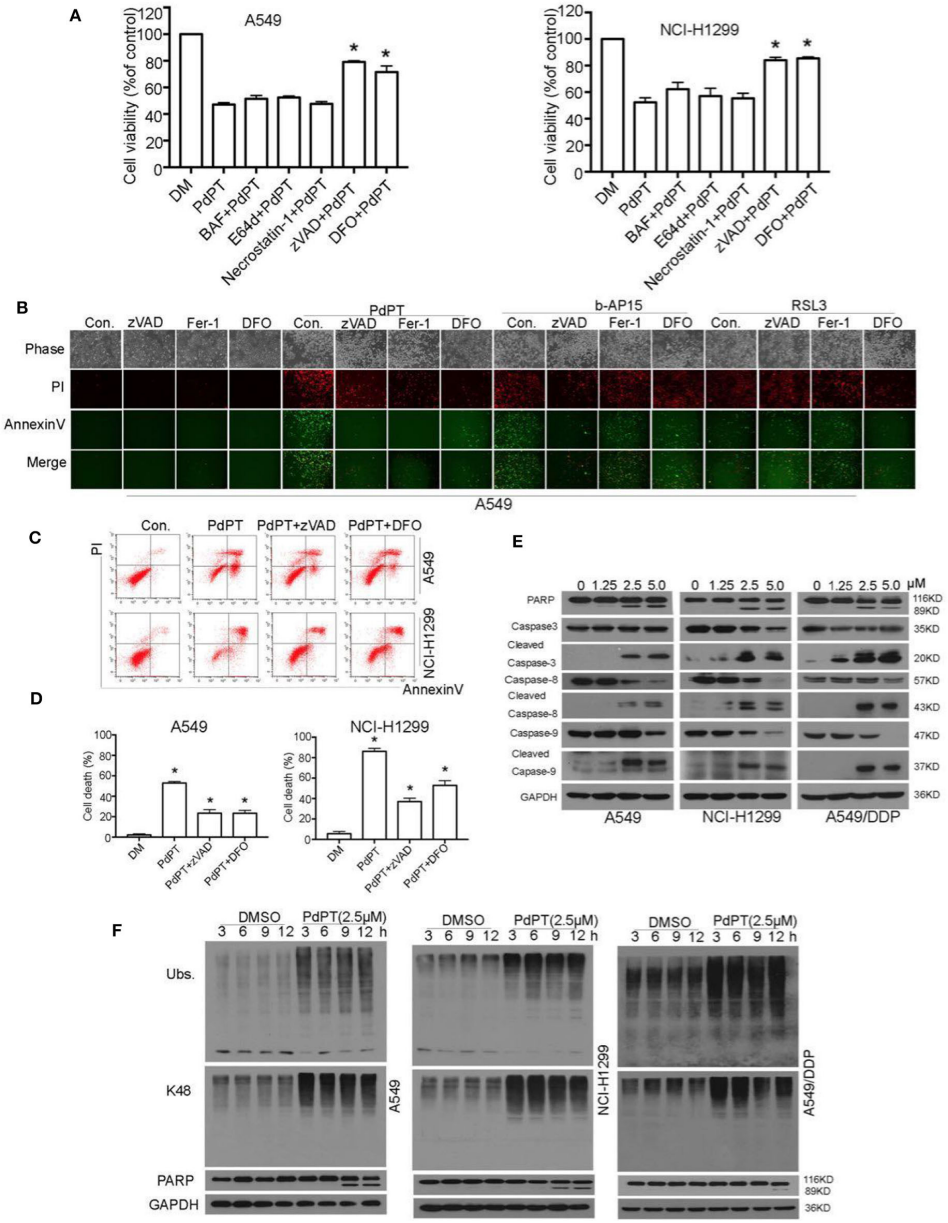
PdPT induces apoptosis and ferroptosis in non-small cell lung cancer cells. **(A)** A549 and NCI-H11299 cells were treated with PdPT (5 μM) in the absence or presence of indicated cell death inhibitors for 24 h. BAF, bafilomycin A1 (100 nM); E64d (10 μM); necrostatin-1 (50 μM); zVAD, z-VAD-FMK (50 μM); DFO, deferoxamine (100 μM); Cell viabilities were analyzed by MTS. Mean ± SD (*n* = 3). *P < 0.05, vs. control. **(B)** A549 cells were treated with PdPT (5 μM), b-AP15 (1 μM) and RSL3 (10 μM) in the presence or absence of z-VAD-FMK (50 μM), ferrostatin-1 (2.5 μM), or deferoxamine (100 μM) for 24 h. The levels of Annexin V- or PI-positive cells were observed using a fluorescence microscope. **(C,D)** A549 and NCI-H1299 cells were treated with PdPT (5 μM) in the presence or absence of z-VAD-FMK (50 μM) or DFO (100 μM) for 24 h. The levels of Annexin V- or PI-positive cells were assayed by flow cytometry. Mean ± SD (*n* = 3). *P < 0.05, vs. control. **(E)** Western blot analysis of cleaved-PARP and cleaved-caspases in A549, NCI-H1299 and A549/DDP cells following treatment with PdPT for 24 h. **(F)** Western blot analysis of indicated proteins in A549, NCI-H1299, and A549/DDP cells following treatment with PdPT for indicated times.

Accumulating evidence shows that necrosis can occur in a highly regulated and genetically controlled manner (23). Although regulated necrosis exhibits different forms such as necroptosis, pyroptosis, ferroptosis, and autophagic cell death, they are characterized by morphological change in plasma membrane rupture with PI staining positive (23). To understand the molecular mechanism of PdPT-induced cancer cell death, we evaluated the effect of various cell death inhibitors on PdPT-mediated cytotoxicity. Interestingly, only the apoptosis inhibitor (z-VAD-FMK) and ferroptosis inhibitor (deferoxamine) partly blocked PdPT-induced cell growth inhibition (Figure 3A). In contrast, the necroptosis inhibitor (e.g., necrostatin-1) and autophagy inhibitor bafilomycin A1 (Baf) and E64D failed to block PdPT-induced cell growth inhibition (Figure 3A). Consistently, flow cytometry and fluorescent microscope analysis confirmed that PdPT-induced up-regulation of annexin V- and/or PI-positive cells was reduced by the apoptosis inhibitor (z-VAD-FMK) and ferroptosis inhibitor (deferoxamine and ferrostatin-1) (Figures 3B–D). z-VAD-FMK, deferoxamine, and ferrostatin-1 also inhibited b-AP15-induced up-regulation of annexin V- and/or PI-positive cells (Figure 3B). As expected, deferoxamine and ferrostatin-1 only reduced RSL3-induced PI-positive cells (Figure 3B). These findings indicate that a mixed type of cell death, including apoptosis and ferroptosis, is required for PdPT-induced cytotoxicity.

Caspases are a family of protease enzymes and the primary drivers of apoptosis (24). Among them, caspase-8 and caspase-9 is implicated in the initiation of extrinsic and intrinsic apoptosis pathway, respectively (22). Activation of caspase-8 and caspase-9 results in the cleavage of caspase-3, the executioner of apoptosis (22). Western blot analysis found that PdPT dose-dependently induced cleavage of caspase-8, caspase-9, and caspase-3 in A549, NCI-H1299, and A549/DDP cell lines (Figure 3E). As expected, the cleavage of PARP (poly(ADP-ribose)polymerase), a classical substrate protein of caspase-3 activation in apoptosis, was increased after PdPT treatment (Figure 3E). These findings indicate that PdPT can trigger both extrinsic and intrinsic apoptosis. Importantly, PdPT-induced accumulation of ubiquitinated proteins was observed before the PARP cleavage, indicating that DUB inhibition is an early event of apoptosis (Figure 3F).

### GPX4 Degradation Is Required for PdPT-Induced Ferroptosis

**Figure 4:**
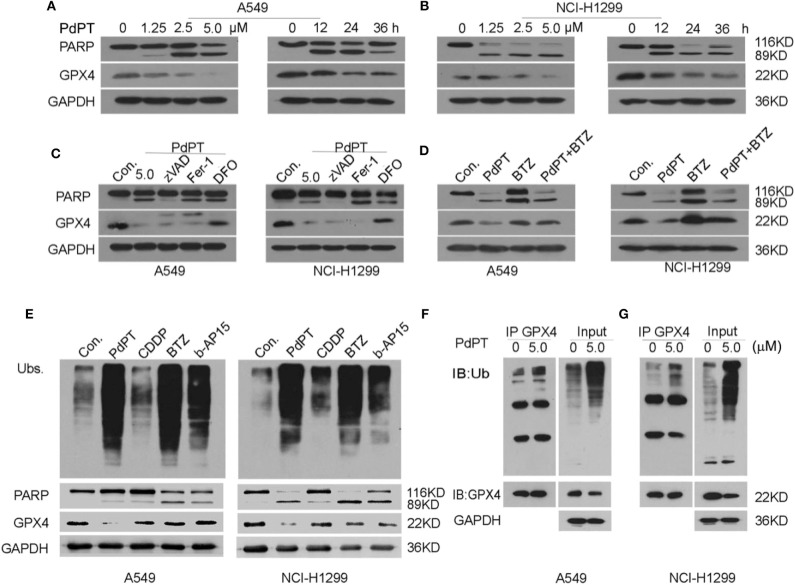
PdPT induces GPX4 protein degradation. **(A,B)** A549 and NCI-H1299 cells were treated with PdPT for indicated does or time and then PARP and GPX4 protein levels were assayed using western blot. **(C)** A549 and NCI-H1299 cells were treated with PdPT (5 μM) in the absence or presence of z-VAD-FMK (50 μM), ferrostatin-1 (2.5 μM), deferoxamine (100 μM) for 24 h. The protein levels of PARP and GPX4 were assayed using western blot. **(D)** A549 and NCI-H1299 cells were treated with PdPT (5 μM) in the absence or presence of bortezomib (BTZ, 100 nM) for 24 h. The protein level of GPX4 was assayed using western blot. **(E)** A549 and NCI-H1299 cells were treated with PdPT (5 μM), CDDP (10 μM), BTZ (100 nM), and b-AP15 (1 μM) for 24 h, and then indicated proteins were assayed using western blot. **(F,G)** Immunoprecipitation analysis of GPX4-Ub complex formation in A549 and NCI-H1299 cells following PdPT treatment for 24 h.

Increased GPX4 degradation has been demonstrated to play an important role in ferroptotic cancer cell death (8, 25, 26). To establish whether PdPT-induced ferroptosis is due to GPX4 degradation, we assayed GPX4 protein levels in A549 and NCI-H1299 cell lines in response to PdPT. Using western blot assays, we found that PdPT induced GPX4 protein degradation in dose- and time-dependent manners, which was parallel to the PARP cleavage (Figures 4A,B). The pan-caspase inhibitor z-VAD-FMK blocked PdPT-induced PARP cleavage, but not GPX4 degradation. Moreover, ferroptosis inhibitors (deferoxamine and ferrostatin-1) did not affect PdPT-induced PARP cleavage (Figure 4C). However, iron-chelating agent deferoxamine, but not lipophilic antioxidant ferrostatin-1, significantly inhibited PdPT-induced GPX4 degradation in A549 and NCI-H1299 cell lines (Figure 4C). Importantly, the proteasome inhibitor bortezomib reversed PdPT-induced GPX4 degradation (Figure 4D), indicating that GPX4 protein degradation is increased by PdPT. Collectively, these findings suggest that PdPT-induced GPX4 proteasomal degradation is iron-dependent, but not caspase- and lipid ROS-dependent.

We next address whether PdPT-induced GPX4 degradation is due to DUB inhibition. As expected, PdPT, bortezomib, and b-AP15 but not CDDP increased the production of total ubiquitinated proteins (Figure 4E). However, only PdPT induced GPX4 degradation in A549 and NCI-H1299 cell lines compared to treatment using bortezomib, b-AP15, and CDDP (Figure 4E). Immunoprecipitation analysis further confirmed that the ubiquitination of GPX4 was increased in response to PdPT (Figures 4F,G). Collectively, these findings indicate a special role for PdPT in the promoting GPX4 protein degradation in ferroptosis.

### *Ex vivo* Effect of PdPT on Primary Monocytes From Acute Myeloid Leukemia (AML) Patients

**Figure 5:**
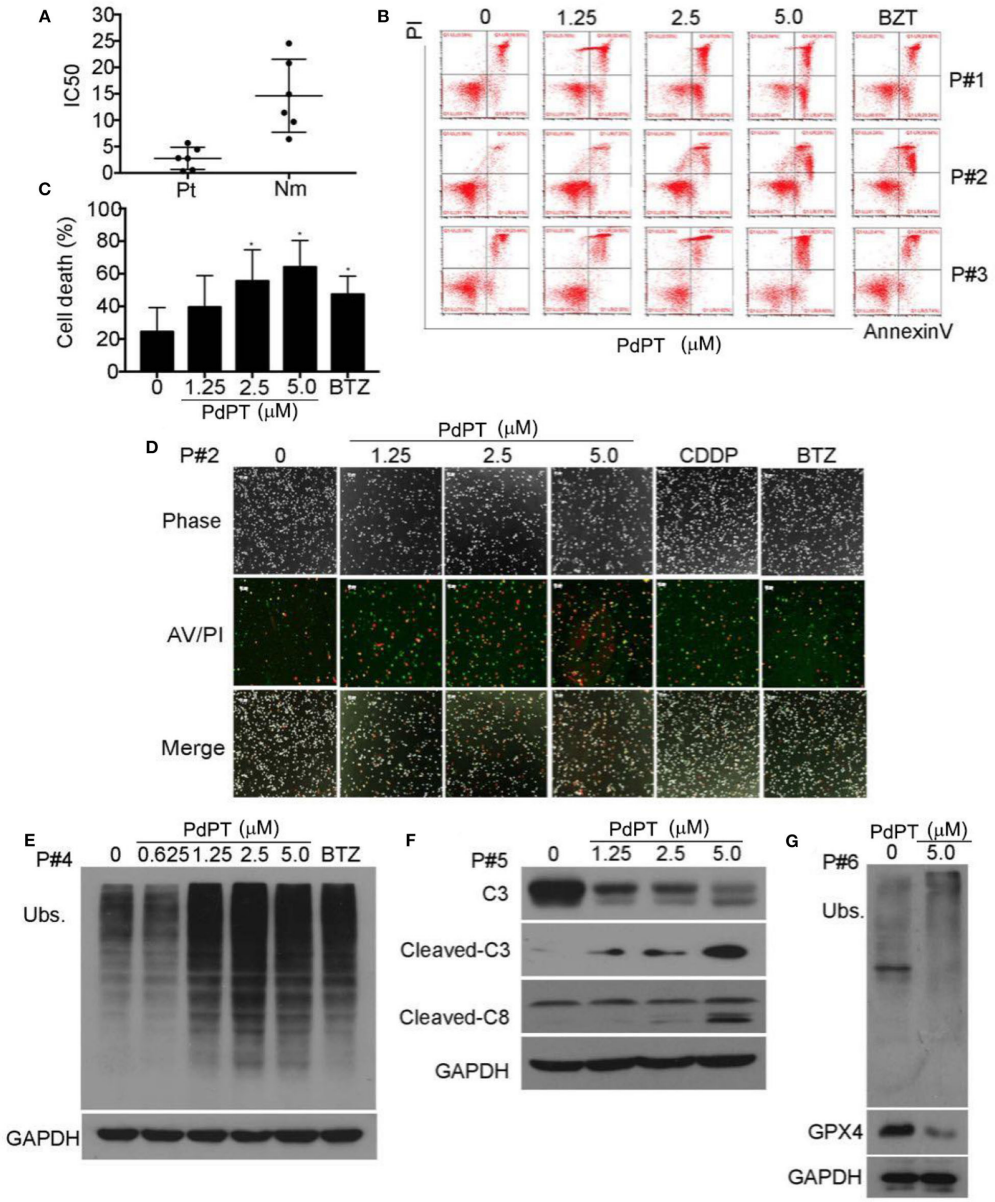
PdPT induces cytotoxity by apoptosis and ferroptosis in cancer cells from AML patients. **(A)** Cancer cells from six AML patients (Pt) and peripheral blood mononuclear cells from six healthy volunteers (Nm) were treated with PdPT at the indicated doses for 24 h, and the cell viability was detected with the MTS assay. The scatter plot of the IC50 values in each group is shown. **(B,C)** Cancer cells from AML patients were incubated with PdPT at the indicated doses for 24 h. Cell death was analyzed with annexin V/PI staining followed by flow cytometry **(B)**, and the results are summarized **(C)**. Mean ± SD (*n* = 3). *P < 0.05, vs. control. **(D)** AML cancer cells were treated with the indicated doses of PdPT, CDDP (5 μM) or BTZ (50 nM) for 24 h, and then cells were stained with Annexin V/PI (A/P) and imaged under a fluorescent microscope. The phase contrast and fluorescent images were taken and merged. Scale bar = 50 μm. Peripheral mononuclear cells of AML cancer cells were treated with PdPT, BTZ (50 nM) for 24 h, followed by detecting total ubiquitinated proteins (Ubs.) **(E)**, caspase-3, cleaved-caspase3, caspase 8 **(F)**, and GPX4 **(G)** with western blotting analyses. GAPDH was used as a loading control. C, control.

Proteasome inhibitors have been proven effective in treating leukemia patients. We next evaluated the *ex vivo* antineoplastic effect of PdPT on bone marrow cells obtained from six patients with AML. Peripheral blood mononuclear cells from six healthy volunteers were used as controls. As displayed in Figure 5A, PdPT decreased the cell viability of primary monocytes from AML patients with average IC50 values of 0.479 μM, while for the peripheral mononuclear cells from 6 healthy volunteers the average IC50 values were 2.372 μM. Next, we found that PdPT induced apoptosis in the monocytes from the AML patients as detected with annexin V/PI staining followed by flow cytometry (Figures 5B,C) or by fluorescence microscopy (Figure 5D). We also found that PdPT markedly induced the accumulation of ubiquitinated proteins (Figure 5E), caspase activation (Figure 5F) and degradation of GPX4 (Figure 5G) in the primary monocytes. These results also show the anti-cancer effect of PdPT in primary human cancer cells.

### Anti-cancer Activity of PdPT *in vivo*

**Figure 6:**
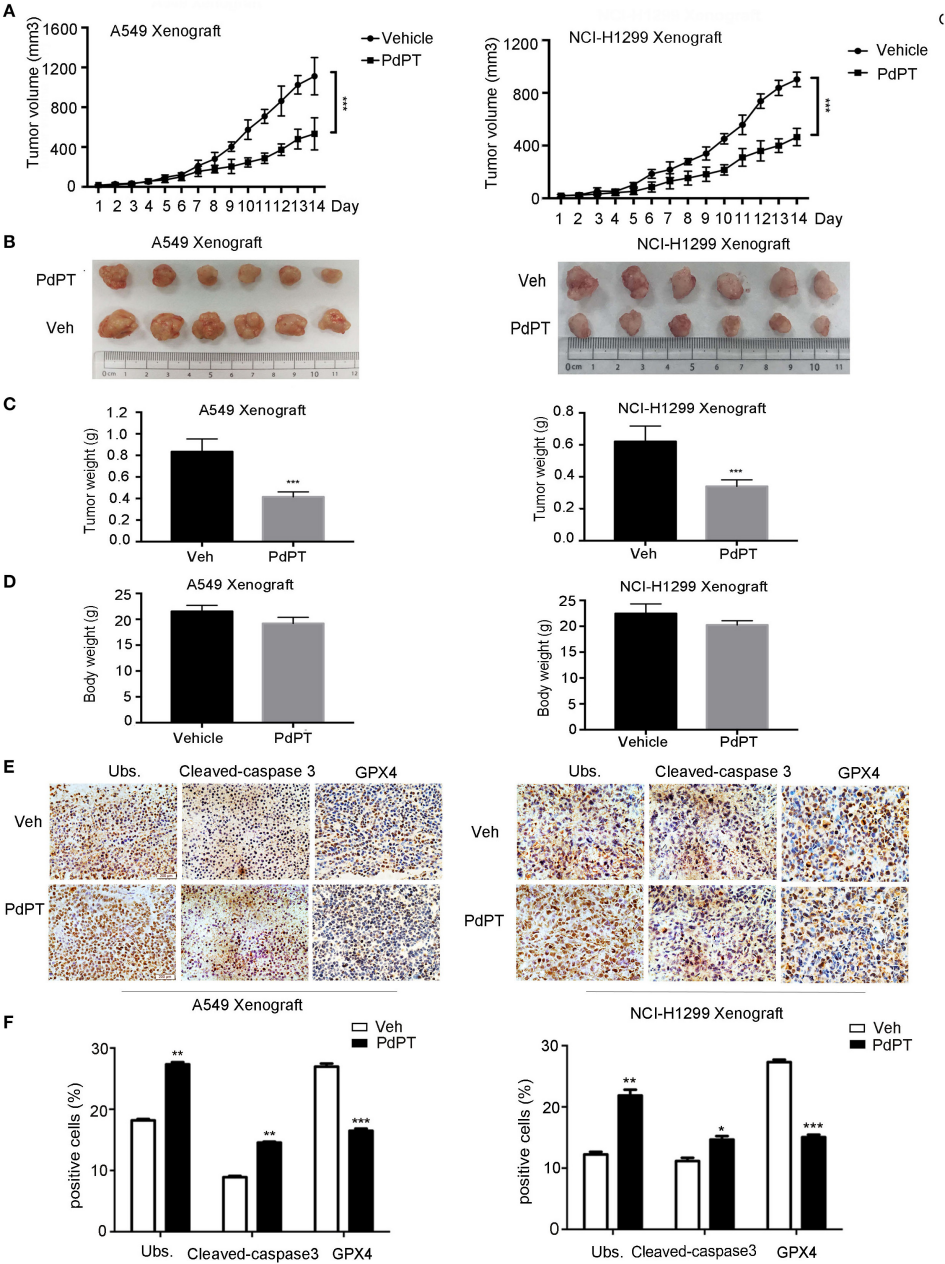
Anti-cancer activity of PdPT *in vivo*. Nude BALB/c mice bearing A549 and NCI-H1299 xenografts tumors were treated with either vehicle (Veh) or PdPT (7.5 mg/kg/day, intraperitoneally) for 15 days when the average tumor size reached at 50 mm^3^. Tumor size was recorded every other day. Tumor size **(A)**, tumor images **(B)**, tumor weight **(C)**, and body weight **(D)** were shown (*n* = 6 mice/group, ***P < 0.001, compared with each control). **(E,F)** Representative image of immunohistochemistry staining and quantification for total ubiquitinated proteins (Ubs), cleaved-caspase 3, and GPX4 in nude mouse tumor tissues (200 ×). Mean±SD. (*n* = 3). *P < 0.05, **P < 0.01, ***P < 0.001, *vs*. Vehicle.

To determine whether suppression of the DUB pathway by PdPT reduces tumor growth *in vivo*, A549 and NCI-H1299 cell lines were implanted into the subcutaneous space of the right flank of nude mice and then treated with PdPT (7.5 mg/kg/day) by intraperitoneal injection. Indeed, the tumor volume, tumor size, and tumor weight were significantly reduced after PdPT treatment compared to the control vehicle (Figures 6A–C). Even though PdPT treatment slightly decreased the body weight, there was no significant difference in the body weight between these two groups (Figure 6D). Moreover, the intratumoral levels of Ubs and caspapse-3 were up-regulated whereas GPX4 was down-regulated after PdPT treatment in these xenograft tumor mouse models (Figures 6E,F). Collectively, these *in vivo* studies support our hypothesis that the DUB inhibitor PdPT suppresses tumor growth partly through induction of apoptosis and ferroptosis.

## Discussion

Lung cancer is the most common cause of cancer death worldwide and new therapy strategies are required for this disease (27). Our current study uncovered that PdPT is a potent and promising anticancer agent for NSCLC, the most common type of lung cancer. We demonstrated that PdPT-mediated DUBs inhibition induces apoptosis and ferroptosis in NSCLC cell lines *in vitro* and *in vivo*. These findings provide new insight into the molecular linking between UPS and regulated cell death in cancer therapy.

Abnormal regulation of the UPS has been known to be involved in the pathogenesis of a variety of human diseases including cancer (28). Inhibition of the proteasome by bortezomib was the first clinical validation of targeting the UPS in cancer therapy (29). However, intrinsic and acquired resistance to bortezomib as well as its toxicities may limit its efficacy (30). Therefore, modulation of upstream components of the proteasome such as DUBs has been investigated as an alternative anticancer strategy (31). DUBs play important role in various events such as metabolism, immunity, survival, and cell death during tumor development and progression (32). A comprehensive understanding of the role of substrate deubiquitylation in the mechanism of 26S proteasome-catalyzed proteolysis will require the assembly of individual DUBs into distinct protein complexes on substrates (33). In this report, we found that PdPT is an inhibitor of multiple DUBs including USP7, USP10, USP14, USP15, USP25 and UCHL5, which contributes to the accumulation of ubiquitinated proteins and subsequent cell death in NSCLC cell lines.

Regulated cell death requires activation of various regulators and effectors (23). The best-studied form of regulated cell death is apoptosis, a complex biochemical process that requires activation of caspases (24). Induction of apoptosis by metal-based compounds has been found to play a major role in the blocking tumor growth (15). Cisplatin is a broad-spectrum anticancer drug and can induce apoptotic cell death through activation of caspase-3 (34). However, the development of cisplatin resistance is the major challenge in the treatment of various solid cancers. The mechanism of acquired cisplatin resistance is multifactor and has been attributed to acquire GSH, an important intracellular antioxidant (35). In this study, we show that targeting DUBs by PdPT can induce a mixed type of cell death in both cisplatin-sensitive and -resistant cancer cells. In addition to caspase-dependent apoptosis, PdPT also induced ferroptosis in NSCLC cell lines, indicating that induction of a mixed type of cell death is worth to be evaluated in cancer therapy in the future.

Our studies also provide the molecular mechanism that underlies the change between GPX4 degradation and ferroptosis. GPX4 degradation is observed in cancer cells in response to classical ferroptosis activators such as erastin, RSL3, and FIN56 (25, 26, 36, 37). Early studies showed that ferroptosis is different from apoptosis, necroptosis, and autophagy in oncogenetic Ras-mutated cancer cells (1). However, recent studies indicate a potential interplay between ferroptosis and autophagy in the regulation of the anticancer activity of ferroptosis activators such as erastin and RSL3 (26, 38–41). Autophagy-mediated ferritin and lipid droplets degradation, and BECN1-mediated system *x*_*c*_− inhibition promotes iron accumulation and GSH depletion in ferroptosis (39–41). However, autophagy is not involved in PdPT-induced ferroptosis, indicating that autophagy has a context-dependent role in cell survival and death (42). The molecular chaperone HSPA5 and HSP90 regulate ferroptosis through modulation of GPX4 degradation in human cancer cells (25, 26). Here, we demonstrated that UPS-mediated GPX4 protein degradation is involved in PdPT-induced ferroptosis in NSCLC cell lines. Thus, the interplay between molecular chaperone and UPS is involved in GPX4 protein regulation to ensure proper function in ferroptosis.

The lipid repair enzyme GPX4 is increasingly recognized as a central negative regulator of ferroptosis by limiting production and cytotoxicity of lipid-oxidation products (7). Conditional knockout of GPX4 in the kidney and T cells can cause lipid peroxidation and subsequent ferroptosis which finally promotes tissue injury and immune dysfunction, respectively (43, 44). In addition to ferroptosis, GPX4 depletion may also be involved in other types of cell death, including necroptosis, pyroptosis and apoptosis, indicating a cell- and tissue-dependent role of GPX4 in regulated cell death (45–47). GPX4 also show variable expression within different types of cancer. GPX4 is up-regulated in colon (48) and liver cancer (49) whereas down-regulated in the pancreas (50), breast (51), kidney (52), and gastric cancer (53). Although increased GPX4 expression promotes drug resistance in tumor therapy, the role of GPX4 in tumorigenesis remains unknown.

## Conclusion

**Figure 7:**
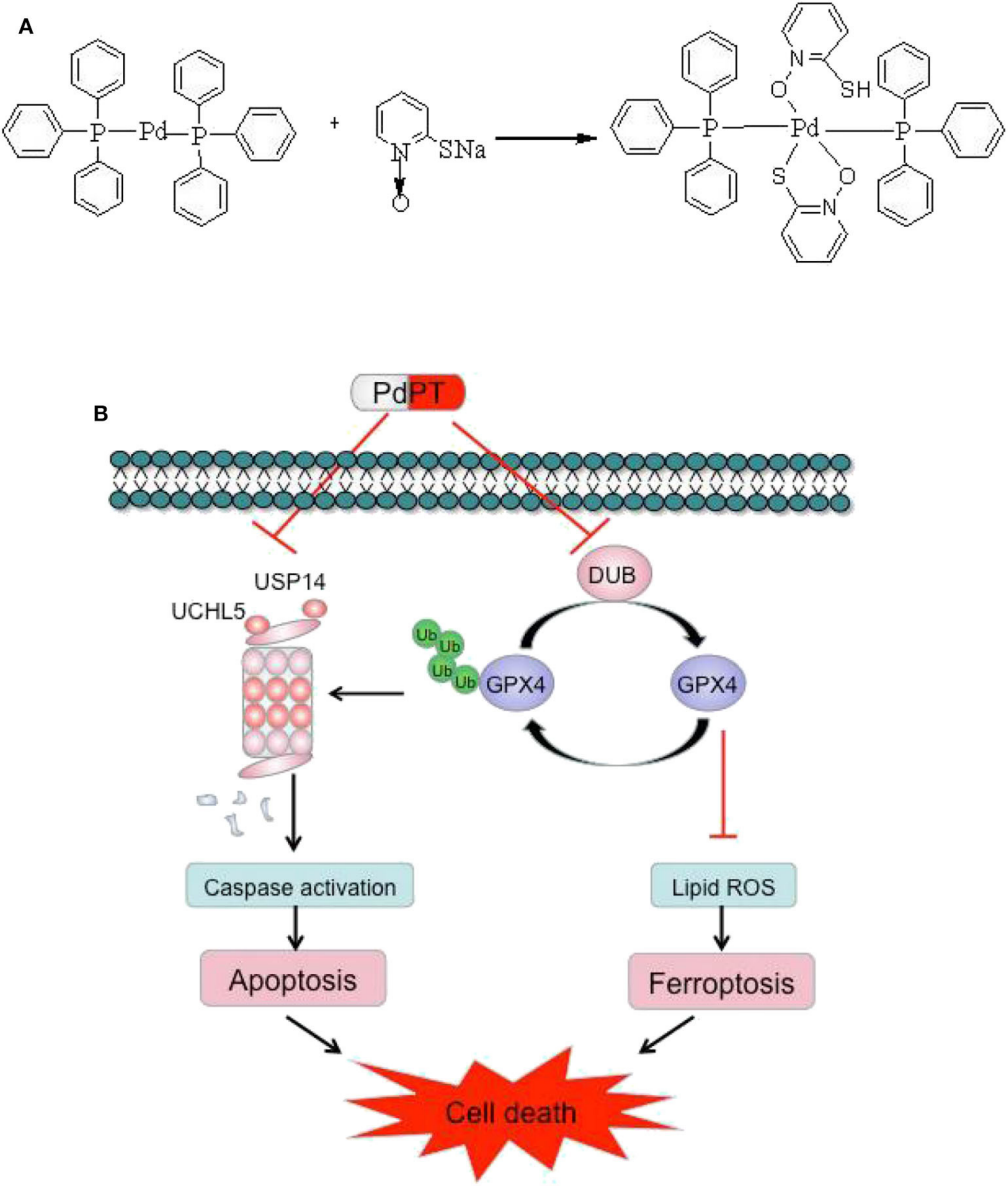
Chemical structure of PdPT **(A)** and a schematic illustration of the proposed model depicting that PdPT induces caspase-dependent apoptosis and GPX4-dependent ferroptosis by inhibition of DUBs **(B)**.

In summary, our results demonstrated that PdPT is a broad-spectrum inhibitor of DUBs that suppresses tumor growth through activation of caspase-dependent apoptosis and GPX4 degradation-dependent ferroptosis. The chemical structure of PdPT and a schematic illustration of the proposed model are illustrated in Figure 7.

## Data Availability Statement

The raw data supporting the conclusions of this article will be made available by the authors, without undue reservation, to any qualified researcher.

## Ethics Statement

The animal study was reviewed and approved by Institutional Animal Care and Use Committee of Guangzhou Medical University (Approval No. GY 2019-113).

## Author Contributions

NZ and JL designed the study. LYan and XC designed the study, analyzed and interpreted the data, and wrote the manuscript. QY, JC, QH, LYan, DY, and JW performed some of the experiments. PZ synthesized the compound. JL and DT revised the manuscript. All authors read and approved the final manuscript.

## Conflict of Interest

The authors declare that the research was conducted in the absence of any commercial or financial relationships that could be construed as a potential conflict of interest.

## Abbreviations

AML: acute myeloid leukemia
AnnexinV-FITC/PI: Annexin V-fluoroisothiocyanate /propidium iodide
Baf: bafilomycin A1
BTZ: Bortezomib
CDDP: cisplatin
DFO: deferoxamine
DMSO: dimethyl sulfoxide
DUBs: deubiquitinating, enzymes
ECL: Enhanced chemiluminescence
FBS: fetal bovine serum
Fer-1: ferrostatin-1
GPX4: glutathione peroxidase 4
HA-Ub-VS: HA-Ubiquitin-Vinyl Sulfone
IC_50_: growth inhibitory concentration 50
NEM: N-Ethylmaleimide
NSCLC: non-small cell lung cancer
PARP: poly(ADP-ribose)polymerase
PdPT: palladium pyrithione
PI: propidium iodide
PS: phosphatidylserine
ROS: reactive oxygen species
Suc-LLVY-AMC: Suc-Leu-Leu-Val-Tyr-aminomethylcoumarin
UPS: ubiquitin-proteasome system
Ubs: Anti-ubiquitin.
